# I see moving people: Expectations drive detection of biological motion in noisy point-light displays

**DOI:** 10.3758/s13423-025-02839-7

**Published:** 2026-01-20

**Authors:** Piotr Szymanek, Magdalena Senderecka, Mateusz Hohol

**Affiliations:** 1 Institute of Religious Studies, Faculty of Philosophy, Jagiellonian University in Krakow, Krakow, Poland; 2 Mathematical Cognition and Learning Lab, Copernicus Center for Interdisciplinary Studies, Jagiellonian University in Krakow, Krakow, Poland; 3 Centre for Cognitive Science, Faculty of Philosophy, Jagiellonian University in Krakow, Krakow, Poland

**Keywords:** Top-down cognition, Agency detection, Predictive processing, Visual noise

## Abstract

While biological motion processing has been extensively studied, little is known about the top-down impact of expectations in this context. We tested whether expectations about the likelihood of encountering a human walker influence the detection of biological motion in point-light displays, particularly when perceptual information is unreliable. Seventy-four participants completed a signal detection task, responding to stimuli featuring either a human walker or scrambled biological motion, each masked with one of four levels of visual noise. Participants were randomly assigned to the high or low expectations group and were told that 75% or 25% of the displays would feature a human walker, although the actual proportion was 50%. Participants in the high expectation group showed a greater tendency to respond “yes,” with the largest group difference emerging at the highest level of noise. These findings suggest that expectations can bias biological motion detection, particularly under conditions of sensory unreliability. The results also support the predictive processing model of agency detection, which proposes that false-positive perceptions of (supernatural) agents arise from expectations combined with ambiguous input.

## Introduction

Patterns of walking movement characteristic of humans—commonly referred to as “biological motion” (Chouchourelou et al., 2013)—are readily recognized by the visual system, even when perceptual information is limited to displays of moving point-lights (Johansson, 1973; Thompson, 2015). Humans not only effortlessly perceive point-lights as marking the joints of a human walker, but also infer a broad range of the walker’s social characteristics (Blake & Shiffrar, 2007). However, despite extensive research on the subject, the influence of top-down processes, including expectations, on biological motion processing remains underexplored.

Here, we addressed this gap by investigating whether the detection of biological motion responds to biased expectations about the likelihood of observing a human walker. The study was motivated by two aims: to broaden our understanding of top-down effects in biological motion processing; and to further test the predictive processing-based model of agency detection (Andersen, 2019).

## Bottom-up and top-down processing of biological motion

While the extent to which perception is shaped bottom-up by sensory input or top-down by expectations, beliefs, or attention remains debated (Clark, 2013; Firestone & Scholl, 2015; Lupyan, 2015; Marr, 1982; Peelen et al., 2024; Pylyshyn, 1999), research on biological motion processing has predominantly focused on identifying the bottom-up conditions for the successful recognition of human walkers. For example, it has been found that short temporal intervals between frames and the presence of dots at the extremities of a walker are crucial for the successful recognition of human gait (Mather et al., 1992). Furthermore, if the stimuli composing the walker are too complex, they inhibit accurate tracking of the movement (Hunt & Halper, 2008). Moreover, studies have shown that biological motion processing is sensitive to inversion (Sumi, 1984), both in humans and in inexperienced chicks (Vallortigara & Regolin, 2006). However, Troje and Westhoff (2006) found that while humans can judge the direction of gait from spatiotemporally scrambled biological motion, this ability also disappears when displays are inverted (cf. Thurman & Lu, 2013). This suggests that the visual mechanism for biological motion perception is triggered by single, orientation-dependent cues (Troje & Chang, 2023). Akin to other mechanisms for quick detection of animate agents, biological motion is hence highly sensitive to bottom-up information (see also Johnson, 2006; Lorenzi & Vallortigara, 2021; Rosa-Salva et al., 2016; Vallortigara, 2012).

Overall, the salience of low-level information, the inversion effect, sensitivity to single cues, evidence for a specialized neural network (Grossman et al., 2005; Pelphrey & Morris, 2006), as well as the fact that newborn children (Simion et al., 2008) and chicks (Vallortigara et al., 2005) respond to biological motion, might suggest that biological motion processing is not only strongly driven by bottom-up information, but also rigid, automatic, hard-wired, and—hence—relatively resistant to cognitive penetration.

In contrast to that view, a handful of studies focused on top-down effects have begun to show that biological motion processing requires attentional resources (Thornton et al., 2002; Nizamoglu & Urgen, 2024) and exploits information from the auditory modality (Brooks et al., 2007). However, only two studies to date have investigated whether biological motion detection can be affected by prior expectations—an effect found in various other perceptual contexts and tasks (see De Lange et al., 2018). First, Uçkan and Urgen (2025) showed that prior auditory cues can facilitate biological motion detection. In their study, participants heard sounds—either footsteps or kicking sounds—that were either congruent or incongruent with the subsequent point-light animation. The authors found that congruent auditory cues sped up the detection of biological motion, although accuracy was unaffected. Second, in a study by Elmas et al. (2025), participants received prior information about the upcoming stimulus—specifically, visual cues indicating the action, emotion, or gender of the walker—and these cues were either congruent or incongruent with the subsequent biological motion. The authors found a congruency effect only for action cues, and only when these cues were moderately reliable (75% validity): participants responded more slowly in incongruent than congruent trials. No such effects emerged for emotion or gender cues. Overall, these studies suggest that the brain utilizes prior information in biological motion processing. The present study extends this line of research by further exploring the robustness of expectations effects in this domain.

### Predictive processing model of agency detection

Our study also addresses a key topic in the cognitive science of religion: agency detection (see Andersen, 2019). The term “agency detection” encompasses a range of cognitive capacities that allow humans to identify intentional agents in their surroundings. These include the perception of self-propulsion, body structure, goal-directed behavior, interactions with other agents, traces of agency left in the environment, and characteristic patterns of movement, including biological motion (see Barrett, 2000; Barrett & Johnson, 2003; Barrett & Lanman, 2008; Lemaire et al., 2022; van Elk, 2013). Such perceptions can lead to representing an object as an intentional agent or a situation as involving one, thereby enabling the ascription of mental states and the prediction of the agent’s future actions.

Cognitive scientists of religion have proposed that our mind is equipped with a Hyperactive Agency Detection Device (HADD), a module that over-detects agency under sensory ambiguity, contributing to the development of supernatural belief (Barrett & Lanman, 2008). However, the HADD model has faced significant criticism (Andersen, 2019; Lisdorf, 2007; Van Leeuwen & van Elk, 2019), and the proposed link between false-positive agency detection and supernatural beliefs lacks strong empirical support (Tratner et al., 2020; van Elk et al., 2016; Van Leeuwen & van Elk, 2019). Most importantly for our study, van Elk et al. (2016) found that supernatural primes did not influence response bias in a biological motion detection task, although an earlier study (van Elk, 2013) reported that paranormal believers are more inclined to false-positive detections of walkers compared to skeptics.

In response to challenges to the HADD model, Andersen (2019) suggested a new, predictive processing-based approach to agency detection. The predictive processing framework views the mind as a prediction-generating machine that continually tests hypotheses against sensory input (Clark, 2013, 2016; Hohwy, 2020). Prediction errors arising from mismatches update the model to improve future predictions. Crucially, the brain estimates whether a prediction matches sensory information through a Bayesian-like inference, weighing the likelihood of the evidence given a prediction against the prior probability of that prediction, with the latter being estimated based on, among other things, knowledge, beliefs, and expectations.

The predictive processing model of agency detection challenges HADD’s claim of a default bias toward false positives, instead proposing that illusory agent detection occurs when (1) the prior probability of encountering agents is high (i.e., they are expected to appear) and (2) sensory data is ambiguous, which increases reliance on priors. Hence, Andersen’s model can explain some instances of encounters with supernatural agents through a combination of expectations (i.e., information about where and when to meet supernatural agents) and low sensory reliability (e.g., dim light, mist, auditory noise)—conditions common in religious practices and environments. These factors have already been found to drive false-positive detections of agents in VR (Andersen et al., 2019) and shape detection of voices (Szymanek et al., 2024). Here, we follow the paradigm used in these studies, aiming to further test the model’s robustness by examining one of the most rudimentary capacities that contribute to agency detection.

### The present study

In the study, we provided participants with explicit information designed to bias their expectations about the likelihood of encountering a human walker in point-light displays. Specifically, we informed them that the proportion of stimuli featuring a human walker was very high or very low, and that the respective chance of encountering a human walker in each individual display was 75% or 25% (henceforth: “high expectations” and “low expectations” group, respectively). In reality, exactly 50% of all animations featured an intact walker, with the rest displaying a scrambled walker. After receiving this information, participants completed a signal detection task (Macmillan, 2002), deciding whether each display featured a walker. Based on their performance, we calculated a response bias measure reflecting their general tendency to respond “yes” or “no.” Furthermore, to test whether expectations drive detection especially under conditions of sensory unreliability, we manipulated the level of noise in the stimuli. Additionally, we measured supernatural beliefs to explore their association with the tendency toward false-positive detection, as suggested by the HADD model.

Our hypotheses were as follows:

- H1: Participants in the high expectations group will exhibit a stronger response bias toward a “yes” response than participants in the low expectations group.
- H2: The effect of expectations on response bias will be more pronounced the higher the levels of noise in the stimuli.

## Methods

### Participants

We collected data from 74 healthy, Polish-speaking participants ages 18–40 years (*M *= 23.12, *SD* = 4.75; 52 women, four other). Participants were compensated with a 50 PLN ($13) gift card. All participants provided written informed consent.

### Materials

#### Signal detection task

The biological motion detection task was implemented in PsychoPy (Peirce, 2007). Participants responded to each animation using a standard QWERTY keyboard, pressing the left or right Control key depending on whether they saw a human walker or not, respectively. They were informed that the walker might appear in different parts of the screen and march left or right, and that they should try to respond even if they were not certain of their response. An exemplary human walker was presented at the beginning of the procedure.

Animations lasted for 2,000 ms and were followed by an additional 2,000 ms response window. A button cue appeared after each animation. All trials began with a 500 ms fixation cross. During training, participants received feedback.

Based on the number of hits and false alarms in the signal detection task (with loglinear correction; see Hautus, 1995), we calculated sensitivity (*d′*: *z*(hits) − *z*(false alarms)) and response bias (*c*: –½(*z*(hits) + *z*(false alarms))). A higher *c* indicated a more conservative response strategy (detecting fewer signals and committing fewer false alarms).

#### Stimuli

We used modified point-light stimuli from the study by van Elk (2013). Fifty percent of the animations featured a walking human avatar made of 12 white points, presented against a black background; the other half featured the same walker, but with all points scrambled spatially across the display. The walker was identical across all animations, and its gender was not specified. Originally, the set consisted of 120 point-light displays with six levels of noise mask, but we trimmed it to 80 animations with four levels of noise mask (24, 48, 96, and 192 scrambled motion points). The walker appeared at five different horizontal locations (−20, −10, 0, 10 and 20 with respect to the center of the screen) and walked left or right. Each animation was presented twice (160 trials total). For the training session, participants saw ten animations each, with stimulus features counterbalanced between groups except for the proportion of intact walkers, which was higher in the high expectations group. Training stimuli featured low levels of noise (12 and 24 points).

#### Questionnaire

Participants filled out the Paranormal and Supernatural Beliefs Scale (PSBS; Dean et al., 2021), which included an attention check. We excluded one participant who failed the attention check from relevant analyses. The questionnaire was translated into Polish by two competent judges (native Polish speakers with high English proficiency) using a forward-translation procedure followed by comparison and discussion to establish the final version. Two terms were culturally adapted to the Polish context (“fairies” to “rusałki,” and “Ouija board” to “plansza spirytystyczna”). The reliability of the scale was satisfactory (*α* = 0.83) and the histogram of mean PSBS scores presented a near-normal distribution, with a slight negative skew. At the end of the questionnaire, we also asked participants whether they guessed what the real aim of the study was and, if yes, at what point of the study they started to suspect that.

### Procedure

Participants were tested in person in the laboratory. They first received general information, which disclaimed only one of the study’s objectives, namely, testing the effect of noise on the detection of human walkers. Participants then began the computerized procedure and were randomly assigned to one of two groups. The testing was conducted on identical Dell OptiPlex 7090 desktop PCs (12th Gen Intel Core i5-12600, 3.30 GHz, 16 GB RAM, Intel UHD Graphics 770) equipped with ASUS BE24EQK 24-in. monitors. The procedure was run in full-screen mode at a 1,440 × 900 resolution. Participants sat comfortably at a distance of approximately 60 cm from the screen. The height of the walker on the display was 734 pixels.

After a brief training session, the participants were told that in the proper task, the number of human animations featuring a walker would vary between participants. In the high expectations group, participants were told that the number of such animations would be very high and that there was a 75% chance that any given animation featured a human walker; in the low expectations group, conversely, the number was said to be very low, with a corresponding 25% chance. Participants then completed the main task in two blocks, separated by a short break. During the break, they were reminded of the expected proportion of animations. Finally, participants completed the questionnaire and were debriefed.

### Data preprocessing

All data processing and analyses were conducted in R (Posit Team, 2025) using the packages *lme4* (Bates et al., 2015), *report* (Makowski et al., 2023), and *emmeans* (Lenth, 2025). We excluded all anticipatory trials (*RT* < 300 ms) and trimmed the data sequentially by *RT*s (±3 *SD*). A linear mixed-effects model (*R*^2^ = .75) revealed that sensitivity decreased at each increasing level of noise: from 24 to 48 (*β* = −0.62, *p* <.001), from 48 to 96 (*β* = −1.57, *p* <.001), and from 96 to 192 (*β* = −2.10, *p* <.001). The dataset, analysis scripts, and materials are available at the Open Science Framework (https://osf.io/v9nm4/).

## Results

### Main analysis

**Fig. 1:**
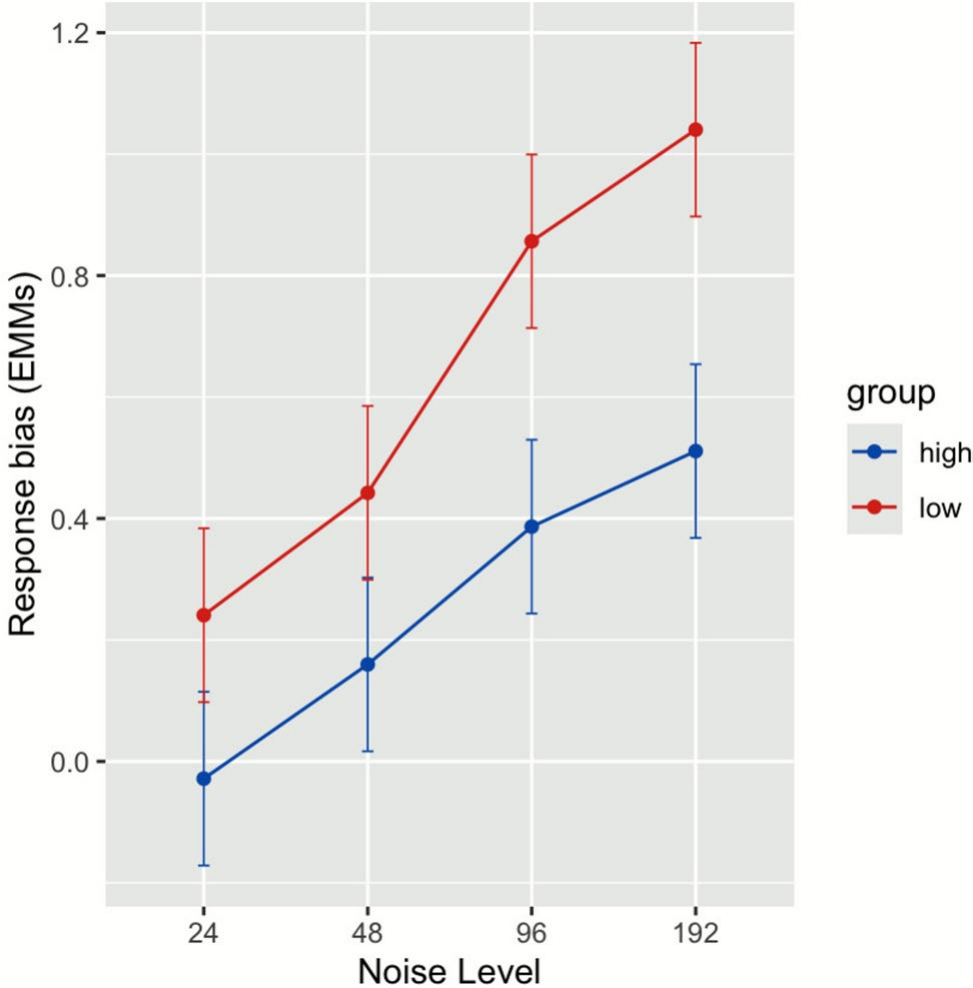
EMMs of response bias by group and level of noise. *Note*. Error bars represent 95% CIs. There was no statistically significant Group × Noise interaction found at noise level 96. (Color figure online)

After testing relevant assumptions, we fitted a linear mixed-effects model with expectations (group) and noise as interacting predictors (*R*^2^ = 0.62). We found a statistically significant main effect of expectations (low-high: *β* = 0.27), *t*(286) = 2.62, *p* =.009, and a main effect of noise at each level: from 24 to 48 (*β* = 0.19), *t*(286) = 2.37, *p* =.019, from 48 to 96 (*β* = 0.42), *t*(286) = 5.22, *p* <.001, and from 96 to 192 (*β* = 0.54), *t*(286) = 6.79, *p* <.001. The group and noise interaction reached statistical significance when noise level increased from 96 to 192 (*β* = 0.26), *t*(286) = 2.31, *p* =.021, but not when it increased from 24 to 48 (*β* = 0.01), *t*(286) = 0.12, *p* =.906, and from 48 to 96 (*β* = 0.20), *t*(286) = 1.79, *p* =.075). A subsequent post-hoc pairwise analysis using estimated marginal means (EMMs) revealed a simple effect of expectations at level 192 (low-high: *β* = 0.53, *t*(195) = 5.16, *p* <.001, *d* = 1.55, and effects sizes diminished with each decreasing level of noise: at 96 (*d* = 1.38), 48 (*d* = 0.83), and 24 (*d* = 0.79). The EMMs are presented in Fig. 1. No ±3 standard deviation outliers of response bias were found. We ran two additional models separately for each block of trials. In the first block, there was no main effect of expectations (*p* =.231), but we found interaction at levels 96 (*p* =.037) and 192 (*p* =.005). Conversely, the analysis for the second block revealed a main effect of expectations (*p* =.003) but no interactions (all *p* values >.05).

Overall, the analysis revealed that: (1) response bias was numerically lower in the high expectations group, pointing to a stronger bias toward a “yes” response and a more liberal response strategy; (2) increasing levels of noise predicted a numerically higher response bias, thus a stronger bias toward a “no” response and a more conservative response strategy; (3) the difference in response bias between groups was most pronounced at the highest level of noise.

### Exploratory analyses

To explore associations between response bias and supernatural beliefs, and their interactions with expectations and noise, we ran three regression models with increasing complexity. First, mean PSBS score alone did not predict response bias (*R*^2^ <.01, *p* = .516). Second, a model including PSBS score and expectations (*R*^2^ = .13, *p* <.001) showed no main effects or interaction (all *p* values >.20). Third, a mixed-effects model with expectations, noise, and PSBS score as interacting predictors (*R*^2^ = .66) revealed statistically significant main effects of expectations (low-high: *β* = .83, *p* = .038) and noise at levels 96 (*β* = 1.30, *p* <.001) and 192 (*β* = 1.50, *p* <.001), but no main effect of PSBS (*β* = 0.26, *p* =.058). The group and noise interacted at levels 96 (*β* = −0.92, *p* = .027) and 192 (*β* = −0.99, *p* =.018). There was no interaction of group and PSBS score (*β* = −0.29, *p* =.127), but PSBS score and noise interacted at levels 96 (*β* = −0.44, *p* =.002) and 192 (*β* = −0.49, *p* <.001). Finally, the three predictors interacted at noise levels 96 (*β* = 0.58, *p* = .004) and 192 (*β* = 0.63, *p* = .002). To explore these interactions, we fitted two additional models with PSBS scores and expectations as predictors, separately for noise levels 96 and 192, but found no significant effects (all *p* values >.05).

Finally, we decided to check for differences in the effect of expectations on response bias between participants who correctly guessed the purpose of the study (and declared guessing it before the half-time of the procedure; *N* = 26) and those who did not. To this end, we separated participants into two groups (guessed vs. did not guess) and ran a linear regression with this grouping variable and expectations as interacting predictors. The model was statistically significant (*R*^2^ = .17, *p* <.001), and there was a main effect of expectations (low-high: *β* = 0.52, *p* <.001), but no main effect of correct guess (guessed-did not guess: *β* = 0.12, *p* = .166). However, we found a statistically significant interaction (*β* = −0.44, *p* <.001) and a post hoc EMMs analysis revealed that expectations affected response bias in participants who did not guess the purpose of the study (low-high: *β* = 0.52, *p* <.001, *d* = −1.04), but not in participants who did (*β* = 0.08, *p* =.864, *d* = −0.16).

## Discussion

This study set out to investigate the top-down effect of prior expectations on the perception of biological motion. We found response bias to be numerically lower in the high expectations group, pointing to a stronger bias toward a “yes” response—hence, our results corroborated H1. Moreover, we observed a stronger effect of expectations at the highest level of noise, partially supporting H2. Still, although the difference between groups was not statistically significant at three lower levels of noise, we observed a consistent pattern: the high expectations group adopted a more liberal response strategy at every level of noise, with increasing noise amplifying the numerical distance between groups. Given the consistency of this trend, we believe it is warranted to claim that the effect of expectations became more pronounced as noise increased.

### Expectations and perception of biological motion

Our findings provide evidence that the perception of biological motion is significantly shaped by top-down expectations. Participants in the high expectations group were more likely to report perceiving a human walker, particularly when visual information was noisy. This aligns with Uçkan and Urgen’s (2025) findings on auditory cues and extends their results by showing that explicitly induced probabilistic expectations alone—without any multisensory congruence—are sufficient to bias perception. Interestingly, our results suggest that prior expectations can not only lead us to detect more biological motion than is present, but also cause us to miss movement that is actually there.

However, our results offer limited insight into the precise computational mechanisms underlying biological motion detection that integrate bottom-up cues and top-down representations. Although these processes can be broadly modeled within a predictive processing framework (see Predictive Processing in Agency Detection section), we cannot determine which visual algorithms are influenced by expectations—those responding to global form or movement, individual cues, or all of the above. Troje and Chang (2023) proposed that our recognition of biological motion is divided into two separate systems: one for detecting cues of biological motion, which governs our attention toward the stimuli, and the other for recognition of global patterns that allow us to discern the agent’s characteristics. We speculate that expectations might make us more vigilant toward individual points that move in a biologically plausible way, potentially explaining why participants in the high expectations group saw more walkers in displays of scrambled biological motion. However, neuroscientific research is needed to determine how exactly expectations influence the detection of biological motion. While studies have examined the neural mechanisms underlying, on one hand, the effects of expectations in perception (see De Lange et al., 2018) and, on the other, detection of animacy and biological motion in humans (e.g., Kim et al., 2011; Servos et al., 2002) and chicks (e.g., Lorenzi et al., 2017, 2024), these lines of research have yet to be integrated.

Finally, we found that the manipulation of expectations affected response bias only in participants who did not guess what the true purpose of the study was. While this exploratory result should be treated carefully, it lowers the possibility that our observations were driven by task demand and suggests that explicit information can affect biological motion processing only if it is considered veridical.

### Predictive processing in agency detection

Our results also provide empirical support for Andersen’s (2019) predictive processing model of agency detection, in which detection of illusory agents can be driven top-down by culturally acquired information combined with sensory unreliability. While the detection of biological motion is one of the most rudimentary capacities enabling us to quickly discern agents in our surroundings, our study shows that it can nonetheless be biased by explicit information, especially under conditions of perceptual noise. This pattern aligns with Andersen et al.’s (2019) and Szymanek et al.’s (2024) findings, thereby generalizing the mechanism to visual agency cues in point-light biological motion. Moreover, we speculate that the effect found by Andersen et al. could have been to some extent driven by false detection of biological motion patterns in the virtual environment, along with perception of other cues, such as voices or footsteps.

Notably, detecting biological motion does not itself require mentalizing or attribution of intentionality, but it often serves as the first step toward recognizing an intentional agent. While our manipulation led participants only to anticipate the presence of walkers—and not, for example, to expect what their actions, goals, or desires were—overall, expecting to see an intentional agent might affect all perceptual and conceptual processes that eventually lead to its full-blown identification. Thus, some expectation-driven detections of illusory agents may begin with a false-positive detection of biological motion, reinforcing the estimated prior probability of encountering an agent. This, in turn, can result in the detection of voices and other agency cues, ultimately leading to attributions of intentions, desires, beliefs, and other mental states.

While our study did not directly address religious experiences, our results support a plausible cognitive mechanism underlying encounters with supernatural agents. If we consider the information provided to participants in the study a proxy for information found “in the wild”—that is, in religious traditions, testimonies of paranormal experiences, cryptozoology, and other—our findings represent a model situation in which expectations induced by culturally transmitted representations, coupled with naturally occurring or human-produced perceptual noise, lead to false detections of agents moving in a biologically plausible way. A possible next step toward linking this model situation to actual extraordinary experiences would be to test whether explicit expectations of *supernatural* agents (e.g., ghosts) could drift participants toward a more liberal response strategy, as perceptual criteria for recognizing a prototypical ghost might be less exact than that of a human.

### Supernatural beliefs and HADD

The HADD model proposes that supernatural beliefs arise, in part, from a cognitive bias toward false-positive agency detection in ambiguous situations (see Andersen, 2019). Our study, which examined one of the perceptual means of identifying agents, did not support this assumption: we found no direct association between supernatural belief scores and response bias, and follow-up regressions revealed no significant interactions between belief strength, noise, and expectations. These results echo other studies reporting mixed or null findings regarding the link between supernatural belief and false-positive detection of agents (Tratner et al., 2020; van Elk et al., 2016; Van Leeuwen & van Elk, 2019).

Our findings also challenge the assumption that the HADD is hair-triggered and produces false-positives whenever sensory evidence is ambiguous, which was said to be its adaptive function (Barrett, 2000, 2007; Barrett & Lanman, 2008; Vallortigara, 2012). In stark contrast to this idea, we found that, regardless of the effect of expectations, increasing noise consistently rendered participants more likely to miss agents rather than to commit false alarms. This result seems to strongly speak against the HADD model, further emphasizing the need to rethink agency detection, in predictive processing terms or other.

## Limitations and conclusions

Several important limitations need to be addressed. First, it should be noted that 70% of our sample were women. Although the human walker was not specifically designed to convey any gender-specific cues, previous research has shown that both the gender of the observers and the perceived gender of the walker can affect the detection of biological motion (Sun et al., 2024). Future studies could examine whether gender differences influence the effects of expectations observed in our study. Second, Vikhanova et al. (2023) found that scrambled biological motion can sometimes be perceived as more “aggressive” than normal walkers. Combined with the proposed effects of threat on agency detection (see, e.g., Szymanek et al., 2025), this could have affected our results. Finally, as the task became increasingly difficult with higher noise levels, participants may have relied more on the provided proportion (75% or 25%) rather than their actual perceptual experience at the highest noise level. Future studies could therefore include a control group informed about the actual 50% proportion. This would provide the estimate of baseline sensitivity and help determine whether the observed bias reflects genuine expectation effects or simply a reliance on base-rate information under high uncertainty.

Summing up, we found that prior expectations can bias the detection of biological motion, particularly under conditions of unreliable sensory information. These results broaden our understanding of biological motion processing and offer further support for the predictive processing model of agency detection.

## Data Availability

The dataset, analysis scripts, and materials are available on the Open Science Framework (https://osf.io/v9nm4/).

## Declarations

### Open practices statement

We report all data exclusions, all manipulations, and all measures in the study. The dataset and experimental procedure are available on the Open Science Framework (https://osf.io/v9nm4/). This study’s design and its analysis were not preregistered.
